# The imbalance in the aortic ceramide/sphingosine-1-phosphate rheostat in ovariectomized rats and the preventive effect of estrogen

**DOI:** 10.1186/s12944-020-01279-7

**Published:** 2020-05-19

**Authors:** Yao Li, Wei Zhang, Junlei Li, Yanrong Sun, Qiyue Yang, Sinan Wang, Xiaofeng Luo, Wenjuan Wang, Ke Wang, Wenpei Bai, Haicheng Zhang, Lihua Qin

**Affiliations:** 1 Department of Cardiology, Peking University People’s Hospital, No. 11 South Avenue, Beijing, 100044 Xi Zhi Men Xicheng District China; 2 Department of Urology, Peking University Fifth School of Clinical Medicine, Beijing, 100730 China; 3 Department of Anatomy and Embryology, Peking University Health Science Center, No. 38, Xueyuan Road, Beijing, 100191 Haidian District China; 4 Department of Stomatology, General Hospital of Armed Police, Beijing, 100039 China; 5 Department of Obstetrics and Gynecology, Shijitan Hospital, Beijing, 100038 China

**Keywords:** Ceramide, Sphingosine 1-phosphate, Ovariectomized rats, Aorta, Estrogen

## Abstract

**Background:**

The prevalence of hypertension in young women is lower than that in age-matched men while the prevalence of hypertension in women is significantly increased after the age of 50 (menopause) and is greater than that in men. It is already known that sphingosine-1-phosphate (S1P) and ceramide regulate vascular tone with opposing effects. This study aimed to explore the effects of ovariectomy and estrogen supplementation on the ceramide/S1P rheostat of the aorta in rats, and to explore a potential mechanism for perimenopausal hypertension and a brand-new target for menopausal hormone therapy to protect vessels.

**Methods:**

In total, 30 female adult SD rats were randomly divided into three groups: The sham operation group (SHAM), ovariectomy group (OVX) and ovariectomy plus estrogen group (OVX + E). After 4 weeks of treatment, the blood pressure (BP) of the rats was monitored by a noninvasive system; the sphingolipid content (e.g., ceramide and S1P) was detected by liquid chromatography-mass spectrometry (LC-MS); the expression of the key enzymes involved in ceramide anabolism and catabolism was measured by real-time fluorescence quantitative polymerase chain reaction (qPCR); and the expression of key enzymes and proteins in the sphingosine kinase 1/2 (SphK1/2)-S1P-S1P receptor 1/2/3 (S1P1/2/3) signaling pathway was detected by qPCR and western blotting.

**Results:**

In the OVX group compared with the SHAM group, the systolic BP (SBP), diastolic BP (DBP) and pulse pressure (PP) increased significantly, especially the SBP and PP (*P* < 0.001). For aortic ceramide metabolism, the mRNA level of key enzymes involved in anabolism and catabolism decreased in parallel 2–3 times, while the contents of total ceramide and certain long-chain subtypes increased significantly (*P* < 0.05). As for the S1P signaling pathway, SphK1/2, the key enzymes involved in S1P synthesis, decreased significantly, and the content of S1P decreased accordingly (*P* < 0.01). The S1P receptors showed various trends: S1P1 was significantly down-regulated, S1P2 was significantly up-regulated, and S1P3 showed no significant difference. No significant difference existed between the SHAM and OVX + E groups for most of the above parameters (*P* > 0.05).

**Conclusions:**

Ovariectomy resulted in the imbalance of the aortic ceramide/S1P rheostat in rats, which may be a potential mechanism underlying the increase in SBP and PP among perimenopausal women. Besides, the ceramide/S1P rheostat may be a novel mechanism by which estrogen protects vessels.

## Introduction

Hypertension is perhaps the most important risk factor for cardiovascular disease. The prevalence of hypertension in young women is lower than that in age-matched men, while the prevalence of hypertension in women increases gradually after the age of 50 (menopause), and is higher than that in men [1]. Furthermore, hypertension in elderly women is more difficult to control than that in age-matched men [1]. Perimenopausal hypertension presents unique hemodynamic characteristics as a result of the increase in arterial stiffness, including greater fluctuations in blood pressure (BP), higher systolic blood pressure(SBP) and pulse pressure (PP), and a decrease in pulse pressure amplification, which makes patients much more prone to target-organ damage [2, 3]. According to the pathogenesis of perimenopausal hypertension (involving sympathetic nervous system activation, renin-angiotensin-aldosterone system activation, imbalances in vasomotor effectors, etc.), an angiotensin-converting enzyme inhibitor (ACEI) or an angiotensin receptor blocker (ARB) combined with β-receptor blockers or verapamil sustained-release tablets is commonly used as the basic antihypertensive treatment. However, effective therapeutic control is still lacking.

Oral menopausal hormone replacement therapy (MHT) is controversial because of its mild hypertensive effect and its potential to increase the incidence of breast cancer. Recently, it has been suggested that improving the timing and the route (oral or subcutaneous) of MHT may achieve the optimal therapeutic effect, but further research is still needed [4, 5]. Perimenopausal hypertension with a high incidence, low control rate, and high mortality/disability rate reduces the quality of life in middle-aged and elderly women.

Sphingolipids are not only integral structural components of cell membranes and organelle membranes, but they also show biological activities in many important biological processes such as cell proliferation, apoptosis, inflammation, and vascular tone regulation [6–9]. Among them, ceramide and sphingosine-1-phosphate (S1P) are the two most well-studied sphingolipid molecules with opposing bioactivities. What’s more, ceramide and S1P can transform into each other, and ceramide-1-phosphate (C1P) has recently been found to regulate the generation of S1P [10]. Ceramide promotes cell apoptosis or senescence through modulating the activity of a series of targets such as p53 [11], while S1P promotes cell survival and proliferation [12–14]. Therefore, the cellular balance of these two sphingolipids contributes to the determination of cell fate, which is called the ceramide/S1P rheostat [7, 15], and the disruption of the rheostat may lead to tumors [16]. Likewise, the presence of long-chain and ultralong-chain ceramides were significantly correlated with vasoconstriction [17, 18], while S1P relaxed the precontracted aorta and mediated the vasodilation effect induced by blood flow [9, 19], and the disruption of the rheostat under pathological circumstances may change vascular tone. Several studies have confirmed a significant correlation between ceramide or S1P and estrogen in breast cancer and plasma [20, 21], but the effect of estrogen on the balance of ceramide/S1P in the aorta has not been reported. The hypothesis is that a low estrogen level may increase the aortic tone by disrupting the ceramide/S1P rheostat, leading to the unique hemodynamic characteristics of perimenopausal hypertension.

In this study, the ovariectomized rat model was used to simulate human menopause, and a sham operation group and an estrogen treatment group were also established. The BPs of the three groups were measured using a noninvasive BP monitoring system. Enzymes and receptors of the S1P signaling pathway were measured by real-time quantitative polymerase chain reaction (qPCR) and immunoblotting, and the aortic sphingolipid content was detected by liquid chromatography-mass spectrometry. The results were then compared between the three groups to determine whether ovariectomy caused an imbalance of the ceramide/S1P rheostat and whether estrogen treatment could prevent the changes in aortic sphingolipid metabolism after ovariectomy, to preliminarily explore the possible mechanism underlying the unique hemodynamic changes observed in postmenopausal women.

## Materials and methods

### Experimental animals and materials

In total, 30 healthy female Sprague Dawley (SD) rats, aged 10–12 weeks, and weighing 220 ± 10 g, were purchased from the Department of Laboratory Animal Science, Peking University Health Science Center, and were approved by the Laboratory Animal Welfare Ethics Committee. Animal experiments were carried out according to the guidelines of the National Institutes of Health and the European Parliament Directive 2010/63/EU on the protection of animals for scientific purposes. The housing and feeding conditions of the experimental animals were as follows: room temperature of 25 °C, 55–65% humidity and a 12 h light/dark cycle; the animals were free to eat soybean-free feed and drink water. After one week of adaption to the new environment, the animals were randomly divided into three groups: The SHAM (sham-operated group), OVX (ovariectomized group), and OVX + E groups (OVX group treated with estradiol valerate). Ten individuals were assigned per group (six of them were used to detect sphingolipid content, and four of them were used to detect the content of related enzymes and receptor proteins). The OVX and OVX + E group were anesthetized with 1% sodium pentobarbital (80 mg/kg intraperitoneal injection) and underwent bilateral ovariectomy, while the SHAM group experienced exploratory laparotomy only. Two weeks after the operation, all rats were injected subcutaneously every day from 8:30 a.m. to 9:30 a.m. for four consecutive weeks. The OVX + E group received estradiol valerate with a dosage of 25 μg/kg/d [22], while the other two groups were administered the same dosage of aseptic sesame oil. The estradiol valerate used was prepared as follows: estradiol valerate (Sigma, E8875) was dissolved in ethanol and then diluted with aseptic sesame oil (Acros, 241,002,500, 10 μg/0.1 mL, 0.25 mL/kg/d, s.c. injection). Four weeks after administration, rats were anesthetized with 1% sodium pentobarbital (80 mg/kg intraperitoneal injection) and euthanized. As for specimen collection, approximately 3–4 mL blood was collected from the heart with 5 mL sterile syringe after thoracotomy, kept in a 37 °C incubator for 30 min, and then centrifuged at 4 °C and 1200 g for 15 min. The upper serum and the whole aorta were then obtained and stored at − 80 °C for further use.

### Hematoxylin and eosin (HE) staining of the vaginal exfoliated cell smear

From day 3 after the operation, vaginal exfoliated cells of all 30 rats were smeared daily for 7 consecutive days to determine whether the model was successfully established. Cotton rods were dipped in normal saline, inserted into the vagina of rats, and gently rotated to obtain the vaginal exfoliated cells. The cells were then evenly smeared on slides. The slides were then dyed with hematoxylin (Coolaber, Beijing, China, SL7050) for 5 min and with eosin (Coolaber, China, SL7060) for 2 min.

### Radioimmunoassay (RIA)

RIA was used to detect the concentration of serum estrogen in three groups. The standards and samples were incubated at 37 °C for 2 h after the addition of the labeled antibody and were then centrifuged at 1200 g for 10 min before being assessed by the radioimmunoassay system. The RIA kit used was obtained from the Beijing Furui Runze Biotechnology Co., Ltd. (Beijing, China). The testing procedure was performed in accordance with the manufacturer’s instructions.

### Real-time quantitative polymerase chain reaction (qPCR)

Aortic tissue was ground with liquid nitrogen, and the messenger RNA (mRNA) was isolated using the TransZol Up Plus RNA Kit (TransGen Biotech Co., Ltd., Beijing, China, Code #ER501) according to the manufacturer’s instructions. Complementary DNA synthesis was carried out with 200 ng mRNA and TransScript II All-in-One First-Strand cDNA Synthesis SuperMix for qPCR (One-Step gDNA Removal) (TransGen Biotech Co., Ltd., Code #AH341). The expression levels of the target genes were determined using the TransStart Top Green qPCR SuperMix (TransGen Biotech Co., Ltd., Code #AQ131) with the Bio-Rad CFX96 PCR system. Relative gene expression was analyzed with the comparative cycle threshold method, and the relative values were standardized to the average expression of glyceraldehyde 3-phosphate dehydrogenase (GAPDH) as the housekeeping gene. The designed primer sets are listed in Supplementary Table 1.

### Western blotting analysis

Protein quantification of sphingosine kinase 1 (SphK1), sphingosine kinase 2 (SphK2), S1P receptor1 (S1P1), S1P receptor 2 (S1P2), and S1P receptor 3 (S1P3) was completed using western blotting analysis, with actin as the internal control. Frozen tissue (25 mg) was chopped on ice with an ultrasonic grinder and suspended in RIPA lysis buffer supplemented with phenylmethane sulfonylfluoride and phosphatase inhibitors and the protein concentration was determined using a BCA Protein Assay Kit (Beyotime Biotechnology, Shanghai, China). The protein samples were separated by electrophoresis on 10% SDS-polyacrylamide gels and transferred onto polyvinylidene fluoride membranes (Millipore, USA). After blocking with 5% skim milk in TBST for 1 h, the membranes were incubated with primary antibodies overnight at 4 °C. Then, the membranes were incubated with HRP-conjugated anti-rabbit secondary antibodies (1:5000 in TBST, Boster Biological Technology, Wuhan, China, BA1054) and incubated at 25 °C for 1 h. After the membranes were washed, the bands were visualized and analyzed using Supersensitive ECL Chemiluminescent Solution (HaiGene Gene Detection Co., Ltd., China, M2301) and the ChemiDoc MP Chemiluminescence imaging system (Bio-Rad, U.S., 170–8280). The primary antibodies used were diluted as follows: SphK1 (Abcam, Cambridgeshire, United Kingdom ab71700, 1:1000, 2 μg/mL); SphK2 (Thermo, Massachusetts, U.S., PA5–51064, 1:1000, 1 μg/mL); S1P1 (Abcam, ab77076, 1:1000,1 μg/mL); S1P2 (Thermo, PA5–23028, 1:1000, 1 μg/mL); and S1P3 (Abcam, ab108370, 1:1000, 1 μg/mL).

### Lipid extraction and liquid chromatography-mass spectrometry

Lipids in the aorta were extracted twice using a modified version of the Bligh and Dyer’s method [23]. In short, we weighed 50 mg of tissue on dry ice and added 900 μl of precooled chloroform: methanol 1:1 (*V/V*) + 10% ultra-pure water to inactivate the sample; then, we smashed the tissue with a bead grinder and put it into a blender for 30 min at 4 °C. Next, 350 μl of ultra-pure water and 300 μl of chloroform were added to the sample. After mixing the tissue and solvents thoroughly, the organic solvent in the lower layer was extracted for the first time. Then, chloroform was added again for a second lipid extraction. The lipid extracts were dried and stored at − 80 °C until further use. For normal-phase liquid chromatography-mass spectrometry (LC/MS), polar lipids were analyzed using an Exion UPLC system in combination with a triple quadrupole/ion trap mass spectrometer (6500 Plus Qtrap; SCIEX) [24]. Individual lipid species with fat acid length ranging from C16 to C24 were quantified relative to the spiked internal standard Cer d18:1/17:0, S1P-d18:1/17:0.

### BP measurement

Five weeks after ovariectomy, we began to measure the BP of the 30 rats at 22:00–24:00 for the next seven consecutive days. The first 6 days were used for adaption training in order to eliminate the impacts of the operation and environment on blood pressure, and as the value became more and more stable, the data were formally collected on the seventh day. BP was measured with the CODA-HT6 Noninvasive BP System (Kent Scientific Corporation, CT, U.S.). The cuff was placed 1 cm from the root of the tail, and affixed the VPR sensor to the tail. The sleeve and VPR sensor were inflated until the occlusive sleeve completely blocked blood flow in the tail, then initiated deflation. When there was blood flowing through the artery, the pressure was registered as the SBP. When the change slope measured by the VPR was the largest, this represented the DBP. Each rat was measured with 10–15 cycles at a time, and the mean value of the last 5 cycles was taken as the final value.

### Data analysis and statistics

All statistical analyses were performed using SPSS 22.0 (IBM SPSS software, Chicago, U.S.) and GraphPad Prism 7 (GraphPad Software, U.S.). All data conformed to a normal distribution with the homogeneity of variance, and are shown as mean ± standard deviation. One-way analysis of variance (ANOVA) and the least significant difference (LSD) test were used to compare the differences between the groups. A value of *P* < 0.05 was considered statistically significant.

## Results

### Successful establishment of the model

#### HE staining of vaginal exfoliated cells in rats

In the SHAM group, the normal estrous cycle was observed, and small, round vaginal exfoliated cells were detected during the nonestrous period, whereas large, polygonal cells with abundant cytoplasm were detected during the estrous period. In the OVX and OVX + E group (before estrogen supplementation), the estrous cycle disappeared, and the vaginal exfoliated cells remained small and round, which was indicative of the success of the bilateral ovariectomy (Supplementary Figure 1A, 1B).

#### Measurement of serum estrogen

The serum estrogen in the OVX group was significantly lower than that in the SHAM group (7.48 ± 2.58 pg/mL vs. 35.76 ± 9.06 pg/mL, *n* = 10, *P* < 0.001), while that in the OVX + E group was significantly higher than that in the OVX group (34.00 ± 6.32 pg/mL vs. 7.48 ± 2.58 pg/mL, n = 10, *P* < 0.001); no significant difference existed between the SHAM and OVX + E groups, which further indicated the success of the model (Supplementary Figure 2).

### Aortic S1P signaling pathway

#### Expression of SphK1/2

**Fig. 1:**
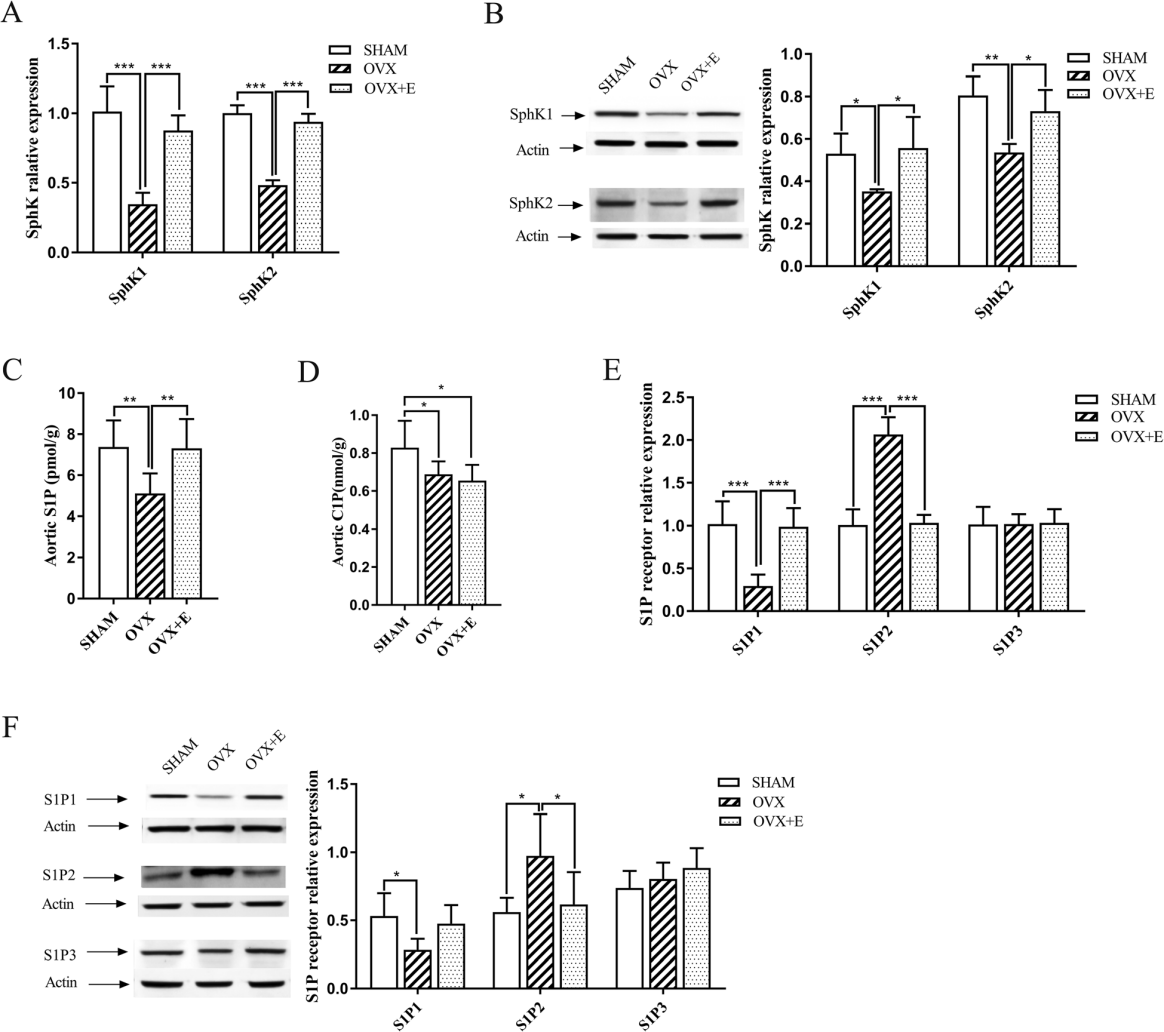
Comparative analysis of S1P signaling between the SHAM group, the OVX group, and the OVX + E group. **a** and **b**: expression of SphK at mRNA (*n* = 4) and protein (n = 4 for SphK1, *n* = 3 for SphK2) levels respectively; **c**: the content of aortic S1P between groups (*n* = 6); **d** content of aortic C1P between groups (n = 6); **e** and **f**: expression of S1P receptors at the mRNA (n = 4) and protein (n = 4 for S1P1, *n* = 5 for S1P2, n = 3 for S1P3) levels respectively. SphK: sphingosine kinase; S1P: sphingosine-1-phosphate; S1P1/2/3: sphingosine-1-phosphate receptor 1/2/3; SHAM group: sham-operated group; OVX group: ovariectomized group; OVX + E group: OVX group treated with estradiol valerate. Data presented as mean ± standard deviation. * *P* < 0.05, ** *P* < 0.01, *** *P* < 0.001

At the mRNA level, the expression of SphK1 (SHAM vs. OVX: 1.01 ± 0.18 vs. 0.35 ± 0.08, *n* = 4, *P* < 0.001; OVX vs. OVX + E: 0.35 ± 0.08 vs. 0.88 ± 0.11, n = 4, *P* < 0.001) and SphK2 (SHAM vs. OVX: 1.00 ± 0.06 vs. 0.48 ± 0.04, *n* = 4, *P* < 0.001; OVX vs. OVX + E: 0.48 ± 0.04 vs. 0.94 ± 0.06, n = 4, *P* < 0.001) in the OVX group was approximately 2–3 times lower than that in the SHAM and OVX + E groups, and no significant difference existed between the latter two groups(*n* = 4, *P* > 0.05) (Fig. 1a).

At the protein level, the expression of SphK1 in OVX was significantly lower than that in the SHAM and OVX + E groups (SHAM vs. OVX: 0.53 ± 0.09 vs. 0.35 ± 0.01, *n* = 4, *P* < 0.05; OVX vs. OVX + E: 0.35 ± 0.01 vs. 0.56 ± 0.15, n = 4, *P* < 0.05), and no significant difference between the latter two groups was found (n = 4, *P* > 0.05); the expression of SphK2 in the OVX group was significantly lower than that in the SHAM group (SHAM vs. OVX: 0.81 ± 0.09 vs. 0.54 ± 0.04, *n* = 3, *P* < 0.01), and the expression of SphK2 in the OVX + E group was significantly higher than that in the OVX group (OVX vs. OVX + E: 0.54 ± 0.04 vs. 0.73 ± 0.10, n = 3, *P* < 0.05). No significant difference existed between SHAM and OVX + E groups (n = 3, *P* > 0.05) (Fig. 1b).

#### Contents of aortic S1P and C1P

The content of aortic S1P in the OVX group was significantly lower than that in the SHAM group (5.11 ± 0.97 pmol/g vs. 7.375 ± 1.29 pmol/g, *n* = 6, *P* < 0.01), while that in the OVX + E group was significantly higher than that in the OVX group (7.31 ± 1.42 pmol/g vs. 5.11 ± 0.97 pmol/g, *n* = 6, *P* < 0.01), and no significant difference existed between the SHAM and the OVX + E groups (Fig. 1c).

Compared with that in the SHAM group, the content of C1P in the OVX and OVX + E groups decreased significantly (SHAM vs. OVX: 0.83 ± 0.14 nmol/g vs. 0.69 ± 0.07 nmol/g, n = 6, *P* < 0.05; SHAM vs. OVX + E: 0.83 ± 0.14 nmol/g vs. 0.65 ± 0.08 nmol/g, n = 6, *P* < 0.05), but no significant difference existed between the OVX and OVX + E groups (Fig. 1d).

#### Expression of S1P receptors

At the mRNA level, the expression of S1P1 (SHAM vs. OVX:1.02 ± 0.26 vs. 0.30 ± 0.13, *n* = 4, *P* < 0.001; OVX vs. OVX + E: 0.30 ± 0.13 vs. 0.99 ± 0.22, *n* = 4, *P* < 0.001)in the OVX group was approximately 3 times lower than that in the SHAM and OVX + E groups, and the expression of S1P2 (SHAM vs. OVX:1.01 ± 0.18 vs. 2.07 ± 0.20, *n* = 4, *P* < 0.001; OVX vs. OVX + E: 2.07 ± 0.20 vs. 1.04 ± 0.09, n = 4, *P* < 0.001) in the OVX group was approximately 2 times higher than that in the SHAM and OVX + E groups; however, no significant change occurred in S1P3 expression (SHAM vs. OVX vs. OVX + E:1.02 ± 0.20 vs. 1.02 ± 0.11 vs. 1.04 ± 0.12, *n* = 4, *P* > 0.05) (Fig. 1e).

At the protein level, the expression of S1P1 protein was significantly lower in the OVX group than in the SHAM group (SHAM vs. OVX: 0.53 ± 0.17 vs. 0.28 ± 0.08, n = 4, *P* < 0.05); compared with that in the OVX group, S1P1 protein expression in the OVX + E group showed an upward trend but no significant difference (OVX vs. OVX + E: 0.28 ± 0.08 vs. 0.48 ± 0.14, n = 4, *P* = 0.07). No significant difference existed between the SHAM and OVX + E groups (n = 4, *P* > 0.05). However, the expression of S1P2 protein was significantly higher in the OVX group than in the SHAM group (SHAM vs. OVX: 0.56 ± 0.10 vs. 0.97 ± 0.31, *n* = 5, *P* < 0.05) or the OVX + E group (OVX vs. OVX + E: 0.97 ± 0.31 vs. 0.62 ± 0.24, n = 5, *P* < 0.05), and no significant difference existed between the latter two groups (n = 5, *P* > 0.05). No significant difference was found in the expression of S1P3 protein between the three groups (SHAM vs. OVX vs. OVX + E: 0.74 ± 0.12 vs. 0.80 ± 0.12 vs. 0.89 ± 0.14, *n* = 3, *P* > 0.05) (Fig. 1f).

### Anabolism and catabolism of aortic ceramide

#### Changes in the mRNA levels of the key enzymes involved in the ceramide anabolic pathway

**Fig. 2:**
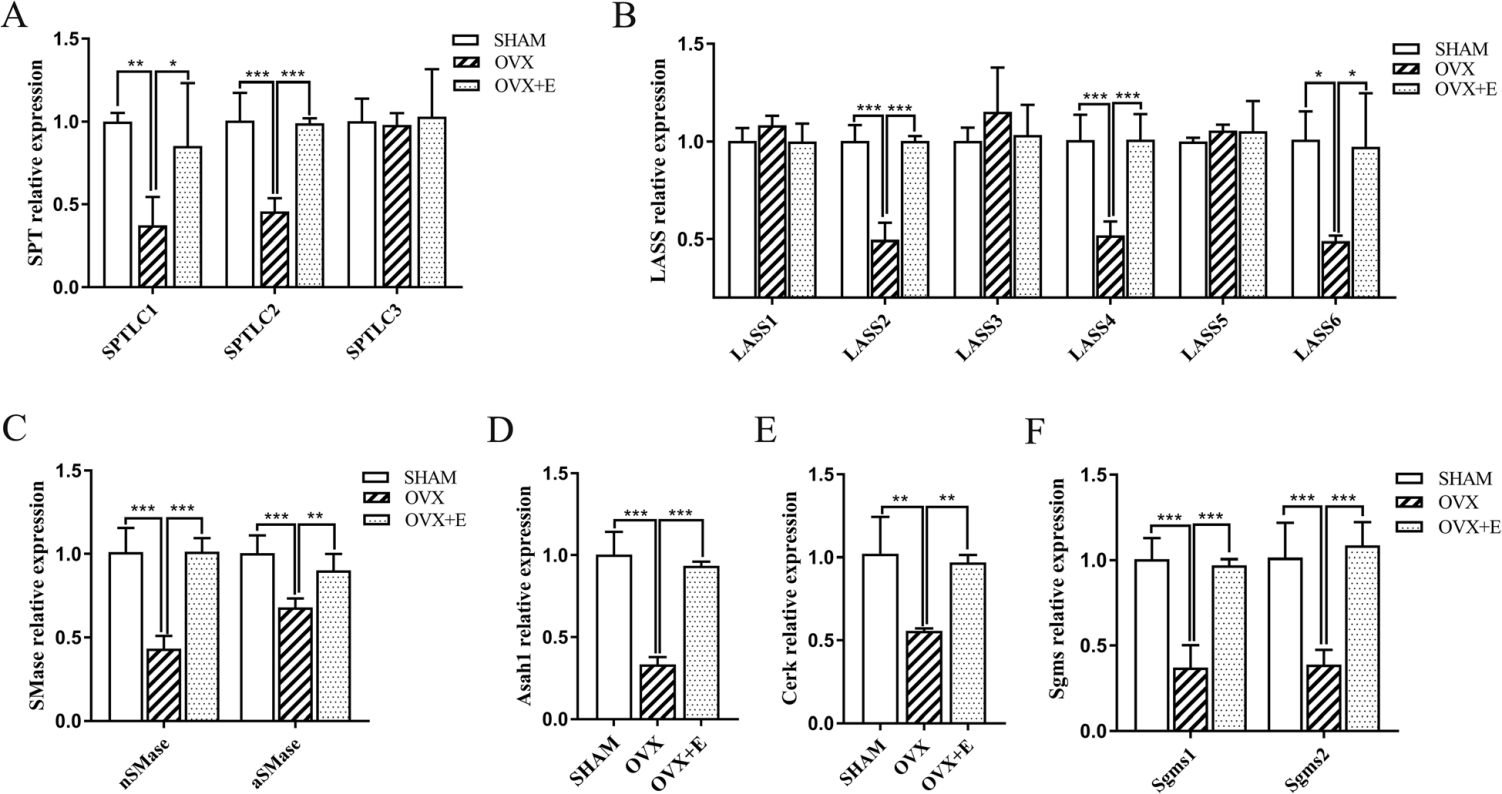
Relative mRNA levels of key enzymes involved in ceramide metabolism. Y-axis represents the expression in each group relative to the mean value in the Sham group. **a**, **b** and **c** represent the anabolic pathways, while catabolic pathways are illustrated in **d**, **e**, and **f**. Cerk, ceramide kinase; Asah, acid ceramidase; Sgms, sphingomyelin synthase; LASS, ceramide synthase; SPTLC, serine palmitoyltransferase long chain base subunit; nSMase, neutral sphingomyelinase; aSMase, acid sphingomyelinase; n = 4 for SPTLC1, LASS4, aSMase, Sgms2,and n = 3 for the rest of the genes. * *P* < 0.05, ** *P* < 0.01, *** *P* < 0.001

Three major pathways are involved in ceramide anabolism; namely the de novo pathway, sphingomyelinase pathway, and salvage pathway [25]. All three subunits of the key enzyme serine palmitoyl-CoA transferase (SPT) in the de novo synthesis pathway were detected. The mRNA levels of SPTLC1 (SHAM vs. OVX: 1.00 ± 0.05 vs. 0.37 ± 0.17, *n* = 4, *P* < 0.01; OVX vs. OVX + E: 0.37 ± 0.17 vs. 0.85 ± 0.38, n = 4, *P* < 0.05) and SPTLC2 (SHAM vs. OVX: 1.01 ± 0.17 vs. 0.46 ± 0.08, *n* = 3, *P* < 0.001; OVX vs. OVX + E: 0.46 ± 0.08 vs. 0.99 ± 0.03, *n* = 3, *P* < 0.001) in the SHAM and OVX + E groups were about 2-fold higher than the levels detected in the OVX group, and no significant difference was observed between the first two groups (*P* > 0.05). The SPTLC3 level was not significantly different among all three groups (SHAM vs. OVX vs. OVX + E: 1.00 ± 0.14 vs. 0.98 ± 0.07 vs. 1.03 ± 0.29, *n* = 3, *P* > 0.05). (Fig. 2a).

**Fig. 3:**
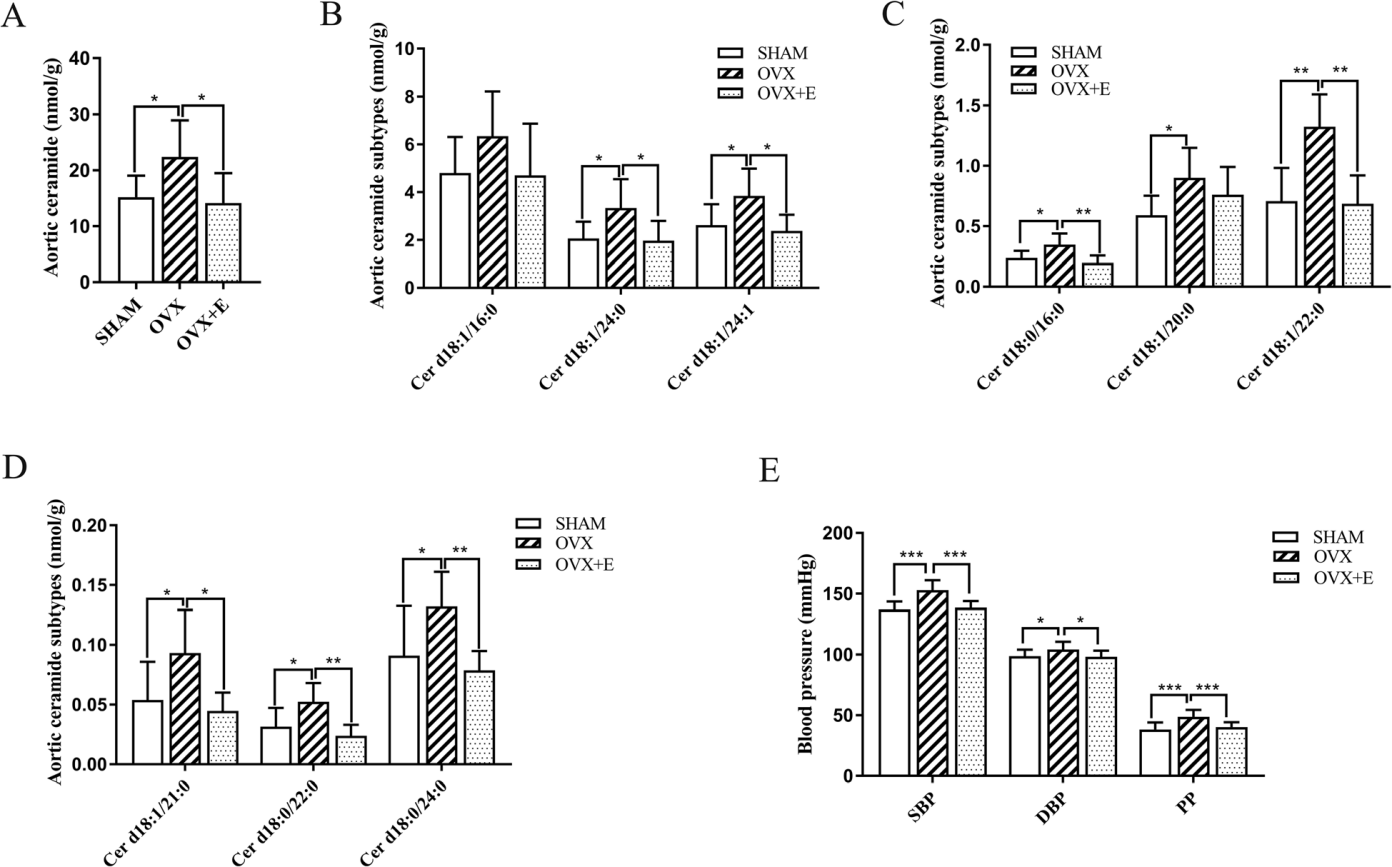
Comparison of total ceramide (**a**), individual ceramide subtypes (**b**), and BP (**c**) among three groups. SBP, systolic BP; DBP, diastolic BP; PP, pulse pressure; SHAM group: sham-operated group; OVX group: ovariectomized group; OVX + E group: OVX group treated with estradiol valerate. n = 6 for ceramide and *n* = 10 for BP, * *P* < 0.05, ** *P* < 0.01, *** *P* < 0.001

Ceramide synthase (LASS) has six subtypes, termed LASS1–6, which are involved in both the de novo and salvage pathways. Of these, the mRNA levels of LASS2 (SHAM vs. OVX: 1.00 ± 0.08 vs. 0.50 ± 0.09, *n* = 3, *P* < 0.001; OVX vs. OVX + E: 0.50 ± 0.09 vs. 1.00 ± 0.03, n = 3, *P* < 0.001), LASS4 (SHAM vs. OVX: 1.01 ± 0.13 vs. 0.52 ± 0.07, *n* = 4, *P* < 0.001; OVX vs. OVX + E: 0.52 ± 0.07 vs. 1.01 ± 0.13, n = 4, *P* < 0.001), and LASS6(SHAM vs. OVX: 1.01 ± 0.15 vs. 0.49 ± 0.03, *n* = 3, *P* < 0.05; OVX vs. OVX + E: 0.49 ± 0.03 vs. 0.97 ± 0.28, n = 3, *P* < 0.05) in the OVX group were decreased to approximately 50% of the levels detected in the SHAM group, and the levels were completely restored after estradiol supplementation. No significant difference was detected in the mRNA levels of LASS1 (SHAM vs. OVX vs. OVX + E: 1.00 ± 0.07 vs. 1.08 ± 0.05 vs. 1.00 ± 0.09, *n* = 3, *P* > 0.05), LASS3 (SHAM vs. OVX vs. OVX + E: 1.00 ± 0.07 vs. 1.15 ± 0.23 vs. 1.03 ± 0.16, *n* = 3, *P* > 0.05), and LASS5 (SHAM vs. OVX vs. OVX + E: 1.00 ± 0.02 vs. 1.06 ± 0.03 vs. 1.05 ± 0.16, n = 3, *P* > 0.05) among the three groups (Fig. 3b), suggesting that ovariectomy could inhibit the de novo synthesis of ceramides with certain lengths of fatty acid chains and that estradiol supplementation could reduce this inhibition. (Fig. 2b).

The level of the mRNA encoding neutral sphingomyelinase (nSMase, one of the key enzymes in the sphingomyelin pathway) was about two-fold higher in the SHAM and OVX + E groups than in the OVX group (SHAM vs. OVX: 1.01 ± 0.15 vs. 0.43 ± 0.08, n = 3, *P* < 0.001; OVX vs. OVX + E: 0.43 ± 0.08 vs. 1.01 ± 0.08, n = 3, *P* < 0.001). The mRNA level of acid sphingomyelinase (aSMase, encoding another key enzyme of the sphingomyelin pathway) was significantly lower in the OVX group than in the SHAM group (SHAM vs. OVX: 1.01 ± 0.11 vs. 0.68 ± 0.05, *n* = 4, *P* < 0.001; OVX vs. OVX + E: 0.68 ± 0.05 vs. 0.90 ± 0.10, n = 4, *P* < 0.01), and estradiol supplementation could prevent this change completely. The effect of both ovariectomy and estrogen supplementation on nSMase was more evident than that on aSMase (Fig. 2c). In summation, ovariectomy could inhibit the expression of specific key enzymes in the ceramide anabolic pathway at the mRNA level, while estrogen supplementation could prevent these changes.

#### Changes in the mRNA levels of the key enzymes involved in the ceramide catabolic pathway

Once generated, ceramides can be directly metabolized by ceramide kinase (Cerk), acid ceramidase 1 (Asah1), or sphingomyelin synthase 1/2 (Sgms1/2) to produce C1P, sphingosine, or sphingomyelin, respectively [10]. The levels of Asah1 (SHAM vs. OVX: 1.00 ± 0.14 vs. 0.33 ± 0.05, *n* = 3, *P* < 0.001; OVX vs. OVX + E: 0.33 ± 0.05 vs. 0.94 ± 0.03, n = 3, *P* < 0.001, Fig. 2d), Cerk (SHAM vs. OVX: 1.02 ± 0.22 vs. 0.56 ± 0.02, n = 3, *P* < 0.01; OVX vs. OVX + E: 0.56 ± 0.02 vs. 0.97 ± 0.04, n = 3, *P* < 0.01, Fig. 2e), Sgms1 (SHAM vs. OVX: 1.01 ± 0.12 vs. 0.37 ± 0.13, n = 3, *P* < 0.001; OVX vs. OVX + E: 0.37 ± 0.13 vs. 0.97 ± 0.04, n = 3, *P* < 0.001, Fig. 2f), Sgms2 (SHAM vs. OVX: 1.02 ± 0.20 vs. 0.39 ± 0.09, *n* = 4, *P* < 0.001; OVX vs. OVX + E: 0.39 ± 0.09 vs. 1.09 ± 0.14, n = 4, *P* < 0.001, Fig. 2f) in the OVX group were 1/2–1/3 of the levels detected in the SHAM group, and no significant difference was observed between the SHAM and OVX + E groups (*P* > 0.05). Thus, ovariectomy inhibited ceramide catabolism, and estradiol supplementation could abolish this effect. Therefore, ovariectomy could also inhibit the catabolism of aortic ceramide.

#### Changes in ceramide levels in the aorta

In general, ovariectomy inhibited the key enzymes involved in the ceramide anabolism and catabolism pathways in parallel. LC-MS was then used to detect the levels of ceramides in the aorta in all three groups, finding that the total amount of ceramides in the OVX group was significantly higher than that in the SHAM group (22.40 ± 6.6 nmol/g vs. 15.20 ± 3.90 nmol/g, *n* = 6, *P* < 0.05). The level of total ceramide total in the OVX + E group was significantly lower than that in the OVX group (14.20 ± 5.30 nmol/g vs. 22.40 ± 6.60 nmol/g, n = 6, *P* < 0.05) and no significant difference existed between the OVX + E and SHAM groups. (Fig. 3a).

As for the ceramide subtype Cer d18:0/16:0, Cer d18:1/21:0, Cer d18:0/22:0, Cer d18:1/22:0, Cer d18:0/24:0, Cer d18:1/24:0, and Cer d18:1/24:1, their levels significantly increased after ovariectomy (n = 6, *P* < 0.05) but decreased to the levels observed in the SHAM group after estradiol supplementation (n = 6, *P* < 0.05). The Cer d18:1/20:0 level significantly increased after ovariectomy (n = 6, *P* < 0.05) and slightly decreased after estradiol supplementation but failed to return to the level observed in the SHAM group (n = 6, *P* > 0.05). The Cer d18:1/16:0 level showed an upward trend in the OVX group and a downward trend after estradiol supplementation, but no significant difference was observed among the three groups (n = 6, *P* > 0.05). (Fig. 3b-d).

### BP changes between the three groups

The SBP of the OVX group was significantly higher than that of the SHAM group (152.90 ± 8.23 mmHg vs. 137.00 ± 6.62 mmHg, *P* < 0.001) and the OVX + E group (152.90 ± 8.23 mmHg vs. 138.60 ± 5.40 mmHg, *P* < 0.001). The DBP of the OVX group was significantly higher than that of the SHAM group (104.20 ± 6.39 mmHg vs. 98.80 ± 5.20 mmHg, *P* < 0.05), and the DBP of the OVX + E group was significantly lower than that of the OVX group (98.30 ± 4.90 mmHg vs. 104.20 ± 6.39 mmHg, *P* < 0.05). The PP of the OVX group was significantly higher than that of the SHAM group (48.70 ± 5.72 mmHg vs. 38.20 ± 5.92 mmHg, *P* < 0.001) and was higher than that of the OVX + E group (48.70 ± 5.71 mmHg vs 40.30 ± 4.00 mmHg, *P* < 0.001). No significant difference existed between the SHAM and OVX + E groups (Fig. 3e).

## Discussion

Ovariectomized rats were used to simulate human menopause to study the effect of ovariectomy on the aortic ceramide/S1P balance and to explore the possible role of the aortic ceramide/S1P rheostat in the occurrence and development of perimenopausal hypertension. After ovariectomy, the vaginal exfoliated cells were smeared to determine whether the ovaries had been completely removed, and then the model was further confirmed by measuring the level of serum estrogen. According to the results, compared with that of the SHAM group, the BP of the ovariectomized rats increased significantly (especially SBP and PP); the levels of the key enzymes of ceramide anabolism and catabolism in the aorta decreased, while the ceramide content increased. The key enzymes of S1P synthesis in the aorta decreased, the content of S1P decreased, the receptors of S1P1 decreased, S1P2 increased, and S1P3 remained unchanged; the ceramide/S1P rheostat was out of balance. Most of the above changes could be prevented by estrogen supplementation.

### The SBP and PP of rats increased significantly after ovariectomy

In postmenopausal women, the aortic pulse index, aortic BP (SBP and PP) and peripheral BP (SBP and PP) are all increased compared with the values measured in young women, which may be related to increased sympathetic activity and greater elastic arterial stiffness caused by decreased estrogen levels [26–28]. In this study, although the SBP, DBP, and PP were all significantly increased in ovariectomized rats, the changes in SBP and PP were more significant, which was consistent with the results of previous animal experiments [29]. The characteristics of perimenopausal hypertension in women suggest the role of the increased stiffness of large arteries in hypertension caused by ovariectomy or menopause. This is also an important reason why this study and many other related studies on perimenopausal hypertension have used the aorta as the target vessel [29, 30].

### The aortic S1P signaling pathway changes after ovariectomy

According to this study, the aortic S1P signal pathway changed significantly after ovariectomy. Firstly, both SphK1 and SphK2 were down-regulated at mRNA and protein levels, indicating that ovariectomy could inhibit the production of aortic S1P at the mRNA and protein levels, which was also confirmed by the reduction of aortic S1P content. It is known that S1P produced by the two subtypes of SphK exerts different functions; S1P from SphK1 is mainly secreted into the extracellular space and participates in the regulation of vascular tone, while SphK2 is mainly involved in cell proliferation and apoptosis [9, 31]. This study focused on the effect of the down-regulation of the SphK1-S1P pathway on vascular tone. Although SphK1 is a cytoplasmic protein, it could be translocated to the cell membrane after phosphorylation by signal-related kinase and the S1P produced near the cell membrane could then be easily transported out of the cell and function in an autocrine or paracrine manner [32]. Cerk/C1P was recently found to be able to promote the translocation of SphK1 [10]. This study found that Cerk/C1P was down-regulated in the ovariectomized group. Therefore, ovariectomy may reduce the SphK1-derived S1P by inhibiting both the expression and function of SphK1. In addition, Guo et al. [21] demonstrated in vitro that after the stimulation of 17β-estradiol (E2), the activity of SphK1, but not SphK2, was markedly increased; the mRNA and protein expression of ATP-binding cassette transporter (ABC) C1, G2 and S1P transporter spinster homolog 2 (Spns2) were significantly elevated, which partly suggested the possible decrease in SphK1 activity and S1P transportation (out of cell membrane) in the aorta of ovariectomized rats. It was reported that S1P could relax the precontracted aorta, and injection of S1P into the aorta could reduce the average arterial BP of rats [19]. Indeed, S1P was found to mediate the flow induced dilation (FID) through NO [9].Therefore, it was supposed that the down-regulation of SphK1 and the concomitant decrease in S1P content after ovariectomy may lead to an increase in aortic tone and stiffness.

S1P regulates vascular tone by binding to the three S1P receptors on the vessel wall. S1P1 and S1P3 (mainly S1P1) in the vascular endothelium mediate vasodilation, while S1P2 and S1P3 in smooth muscle cells mediate a vasoconstrictive effect [17, 33–35]. Therefore, the regulation of S1P on vascular tone depends on the comprehensive effect of its receptors. Few studies have reported the effect of estrogen on the expression of S1P receptors in blood vessels for now. Hemmings et al. [36] found that estrogen treatment could increase the expression of the S1P1 receptor in the mesenteric artery of aging rats. However, the tone-regulation effects of S1P signaling on the resistance artery and on the volume artery were quite opposite [19, 37]; thus, the results for the mesenteric artery could not directly predict the changes in S1P1 in the aorta. Furthermore, the effect of menopause or estrogen on the expression of S1P2/3 in blood vessels is not yet clear. This experiment found that, compared with that in the SHAM group, the expression of aortic S1P1, which mediated the vasodilator effect, decreased in the OVX group (consistent with the findings of Hemmings et al.), while the S1P2-mediated vasoconstrictor effect increased, and S1P3 remained unchanged. Therefore, the combined effect of S1P receptor changes was to increase the aortic tone after ovariectomy. It was reported that S1P regulated vascular tone through S1P-S1P1-eNOS-NO-mediated vasodilation in the endothelium [9], and S1P-S1P2/3-RhoA/ROCK-MLCP-pMLC-mediated vasoconstriction in smooth muscle cells [38]. Interestingly, studies have found estrogen could attenuate the contraction of the aorta ring by inhibiting the RhoA/ROCK-MLCP-pMLC pathway [39] and MLC17a expression in the aorta of ovariectomized rats was found to be down-regulated [40]. In addition, NO activity in the aorta of ovariectomized rats was also decreased [41]. Therefore, it was supposed that supposed the possible vasoconstriction effect of the S1P signaling pathway on aortic tone under the condition of low estrogen was mediated by the down-regulation of S1P-S1P1-eNOS-NO and the up-regulation of S1P-S1P2/3-ROCK-MLCP-pMLC, which needs further study and confirmation.

Kerage et al. found that S1P could also affect the interaction between circulating vasoconstrictors and smooth muscle cells by regulating the permeability of endothelial cells and thus regulating the vascular tone [35]. The S1P and S1P1 receptor agonist FTY720 could rapidly reduce the pulmonary edema and vascular permeability caused by vascular endothelial growth factor [42], indicating that the S1P-S1P1 signaling pathway has a protective effect on the functional integrity of the endothelial barrier. This study found that after ovariectomy, the activity of the signaling pathway of S1P-S1P1 in rat aorta decreased, which may be related to the impairment of vascular endothelial barrier function. Given that ovariectomy could increase the permeability of the blood-brain barrier via the redistribution of tight junction proteins [43], and estrogen could play a protective role in the endothelial barrier through a variety of mechanisms [44], it was supposed that the down-regulation of S1P-S1P1 in the aorta of ovariectomized rats may be another mechanism underlying the increase in vascular endothelial permeability caused by the decrease in estrogen. Since the plasma concentration of S1P is significantly higher than that in interstitial tissue [45], plasma S1P may exert a vasoconstrictive effect by contacting the S1P2 and S1P3 receptors on vascular smooth muscle through the damaged endothelial barrier along the concentration gradient after ovariectomy.

### Changes in ceramide metabolism in the rat aorta after ovariectomy

The correlation between estrogen and ceramide has mainly been reported in breast cancer, serum, plasma and other tissues [46–49]. However, the effect of estrogen or ovariectomy on ceramide metabolism in vessels has never been reported. This study first explored the changes in ceramide anabolism and catabolism between the SHAM, OVX, and OVX + E groups. As long-chain and ultralong-chain ceramides are more abundant in blood vessels, and they showed abnormal levels in various vascular diseases, such as hypertension and coronary heart disease [50–52], this study focused mostly on the changes in long-chain and ultralong-chain ceramides in the aorta.

According to these results, the mRNA levels of key enzymes involved in the anabolism and catabolism of aortic ceramide were down-regulated after ovariectomy, indicating the inhibition of aortic ceramide metabolism by ovariectomy. There are six different subtypes of LASS, the key enzyme involved in the de novo pathway and remedy pathway, and each catalyzes the synthesis of ceramide with certain fatty acid chain lengths. The study showed no significant difference in the levels of LASS1, LASS3, and LASS5, while the mRNA levels of LASS2, LASS4 and LASS6 were significantly down-regulated, indicating ovariectomy may only affect the content of ceramide with certain fatty acid chains lengths. As LASS2, LASS4 and LASS6 were found to be significantly correlated with Cer-16, Cer-18, Cer-20, Cer-24 and Cer-24:1 in breast cancer tissue [47], it was supposed that ovariectomy had an effect on these long- and ultralong-chain-ceramides in the aorta. Therefore, the study further detected the content of ceramide in the aorta by LC-MS. The results showed that the total amount of ceramide increased, and the contents of certain subtypes were also significantly increased, such as Cer d18:0/16:0, Cer d18:1/20:0, Cer d18:1/21:0, Cer d18:0/22:0, Cer d18:1/22:0, Cer d18:0/24:0, Cer d18:1/24:0, and Cer d18:1/24:1. Based on the decrease of key enzymes involved in ceramide anabolism and catabolism in parallel and the increased ceramide content in the aorta, it was supposed that the inhibition by ovariectomy of ceramide catabolism was much greater than that of anabolism, thus resulting in the accumulation of ceramide in the aorta. Similar trends have been reported in other studies as well. For example, Wegner et al. [46] found that the mRNA levels of key enzymes involved in sphingolipid catabolism and anabolism in breast cancer cells overexpressing GPER1 increased in parallel, while in contrast, the final sphingolipid content decreased.

This study found that some long- and ultralong-chain ceramide subtypes accumulated in the aorta of ovariectomized rats, and these subtypes have been reported to be associated with hypertension in several studies. Spijkers et al. [17, 50]found the levels of ceramide (Cer-14, Cer-16, Cer-18:1, Cer-18, Cer-20, Cer-22 and Cer-24:1) in the aorta of spontaneously hypertensive rats (SHR) were significantly higher than those of normal hypertensive rats (WKY), while the levels of Cer-14, Cer-16:dh, Cer-20, Cer-22, Cer-24, Cer-24:1, Cer-26 and Cer-26:1 were significantly reduced after the application of valsartan and hydralazine, indicating that the accumulation of long- and ultralong-chain ceramide subtypes in the aorta may be closely related to the occurrence and development of hypertension. In vitro experiments further suggested that ceramide induced vasoconstriction by iPLA2, cyclooxygenase-1 and thromboxane synthase [50]. In addition, one clinical study found a significant correlation between plasma ceramide (Cer d18:1/16:0) and hypertension in postmenopausal women [48].

Ceramide could lead to the dephosphorylation of eNOS and could reduce NO production through a variety of mechanisms [53–55]. A positive feedback amplification effect between ceramide and reactive oxygen species (ROS) in vascular tissue has been reported [56], and ROS could act as a second messenger to mediate or interfere with the vasorelaxation of NO, prostacyclin, angiotensin II, endothelin-1, TXA2 and other major vasoactive factors [57]. In addition, long-term chronic ceramide exposure could change the vasoactive mediators of blood flow-induced relaxation from NO to harmful ROS, resulting in abnormal FID and increased vascular stiffness [18, 58]. Recently, Vidal-Gomez et al. found that the production of superoxide in the aorta of ovariectomized mice increased while the activity of NO decreased [41]. Overall, it was supposed the increased long- and ultralong-chain ceramide subtypes in the aorta of ovariectomized rats may increase aortic tone via a decrease in NO and an increase in ROS, which needs to be further confirmed.

### Imbalance of the ceramide/S1P rheostat

Long-chain and ultralong-chain ceramides mediate the increase in vascular tone through various mechanisms, while the S1P pathway mediates vasodilation in the aorta, and the two restrict each other in the regulation of the aortic tone via the ceramide/S1P rheostat [15]. According to these results, long-chain and ultralong-chain ceramides were increased in the aorta of ovariectomized rats, while the vasodilation effect of the S1P signaling pathway transformed into vasoconstriction after ovariectomy; thus, the disruption of the ceramide/S1P rheostat may eventually lead to an increase in aortic tone.

### Estrogen supplementation could prevent most of the above described changes

The cardiovascular protective effect of estrogen has been widely confirmed by many clinical and basic experiments [59, 60]. This study explored the vascular protective effect of estrogen from the perspective of sphingolipid metabolism and found that subcutaneous estrogen supplementation could prevent an imbalance in ceramide/S1P in the aortas of ovariectomized rats, which further indicated that a low estrogen level may be the main mechanism underlying the changes in aortic sphingolipid metabolism after ovariectomy, instead of progestogen or other hormones. However, this requires further study and confirmation.

Interestingly, the protein expression of S1P1 and the content of Cer d18:1/20:0 and C1P in the OVX + E group showed certain changes when compared with the levels in the OVX group, but no significant difference existed between those two groups. This lack of significance may be due to the participation of progesterone and androgen in addition to that of estrogen.

Although the cardiovascular protective effect of estrogen has been confirmed, the clinical application of MHT is still controversial. Recently, the French Association of Hypertension stated that oral MHT may slightly increase hypertension, but that subcutaneous injection of MHT has better antihypertensive effects. In addition, it suggests that patients with perimenopausal hypertension and significant menopausal symptoms who are under 60 and within 10 years of menopause be prescribed with subcutaneous MHT after full evaluation of the benefits and risks [5]. This study found that subcutaneous injection of estrogen could reverse hypertension caused by ovariectomy, consistent with the results of previous animal experiments [61, 62] and providing further support for clinical application of MHT in patients with perimenopausal hypertension.

### Study limitation

This study preliminarily revealed the effects of ovariectomy and estrogen supplementation on ceramide/S1P rheostat in the rat aorta, which explored a new mechanism for perimenopausal hypertension and showed certain clinical significance. However, it remained unclear that how the ceramide/S1P rheostat regulated vascular tone and how ovariectomy and estrogen supplementation affected the rheostat. This study raised theoretical hypothesis based on the current results and other studies, yet there is still a broad space for further exploration and research.

## Conclusions

Here, this study assessed the effects of ovariectomy and estrogen treatment on the aortic sphingolipid rheostat for the first time. The results suggested: 1. Ovariectomy increased BP in rats, especially SBP and PP. 2. Ovariectomy resulted in the imbalance of the aortic ceramide/S1P rheostat in rats. The accumulation of long-chain ceramide associated with increased aortic tone and vasoconstrictive transformation of the S1P signaling pathway suggested that the imbalance in the sphingolipid rheostat may mediate the increase in aortic tone, which may be a potential mechanism underlying the increase in SBP and PP among perimenopausal women. 3. Estrogen intervention prevented most changes in aortic sphingolipid metabolism and BP after ovariectomy, suggesting that the ceramide/S1P rheostat may be a new mechanism by which estrogen protects vessels.

### Potential clinical value

Research on bioactive sphingolipids has led to the discovery of beneficial drugs for use in treating diseases, such as the S1P receptor modulator used for treating multiple sclerosis [63]. Zhang et al. [55] found that pharmacological inhibition of de novo ceramide synthesis, using the SPT inhibitor myriocin, and heterozygous deletion of dihydroceramide desaturase prevented hypertension in mice after high-fat feeding, and Swendeman et al. [64] reported that the administration of a soluble carrier of S1P, ApoM-Fc, reduced blood pressure in hypertensive mice. The two researches respectively suggested the potential value of reducing ceramide accumulation and promoting S1P signaling in mitigating ordinary hypertension. According to this experiment, the disruption of the aortic ceramide/S1P rheostat may account for the increased SBP and PP in perimenopausal women. Therefore, the aortic ceramide/S1P rheostat may be a brand-new therapeutic target for effective management of perimenopausal hypertension.

## Supplementary information

**Additional file 1: Figure S1.** HE staining of vaginal exfoliated cells. A: estrus period, the exfoliated cells were large, polygonal cells with abundant cytoplasm, as is indicated by the black arrow. B: nonestrus (estrus interval) period, the exfoliated cells were small and round with little cytoplasm as is indicated by the black arrow.

**Additional file 2: Figure S2.** Comparative analysis of the concentration of serum estrogen between the SHAM group, the OVX group, and the OVX + E group. SHAM group: sham-operated group; OVX group: ovariectomized group; OVX + E group: OVX group treated with estradiol valerate. Data presented as the mean ± standard deviation, *n* = 10. * *P* < 0.05, ** *P* < 0.01, *** *P* < 0.001.

**Additional file 3: Table S1.** Primer sets of targeted genes.

## Data Availability

Not applicable.

## Abbreviations

ROS: Reactive oxygen species
PP: Pulse pressure
DBP: Diastolic blood pressure
SBP: Systolic blood pressure
ARB: Angiotension receptor blocker
ACEI: Angiotensin converting enzyme inhibitor
MHT: Menopausal hormone therapy
S1P: Sphingosine-1-phosphate
Cer: Ceramide
C1P: Ceramide-1-phosphate
SPT: Serine palmitoyl transferase
a/nSMase: Acid/neutral sphingomyelinase
Cerk: Ceramide kinase
Asah1: N-acylsphingosineamidohydrolase1
SphK1/2: Sphingosine kinase1/2
S1P1–5: Sphingosine-1-phosphate receptor 1–5
Sgms1/2: Sphingomyelin synthase1/2
CerS/LASS: Ceramide synthases/longevity assurance
MLCK: Myosin light chain kinase
MLCP: Myosin light chain phosphatase
eNOS: Endothelial nitric oxide synthetase
FID: Flow induced dilation
HE: hematoxylin-eosin staining
LC-MS: Liquid chromatography-mass spectrometry
qPCR: Quantitative polymerase chain reaction
WB: Western blotting
