# Supplementary figures and images for: The imbalance in the aortic ceramide/sphingosine-1-phosphate rheostat in ovariectomized rats and the preventive effect of estrogen
Source: Lipids Health Dis. 2020 May 19;19:95. doi: 10.1186/s12944-020-01279-7 (PMC7236922; doi:10.1186/s12944-020-01279-7)

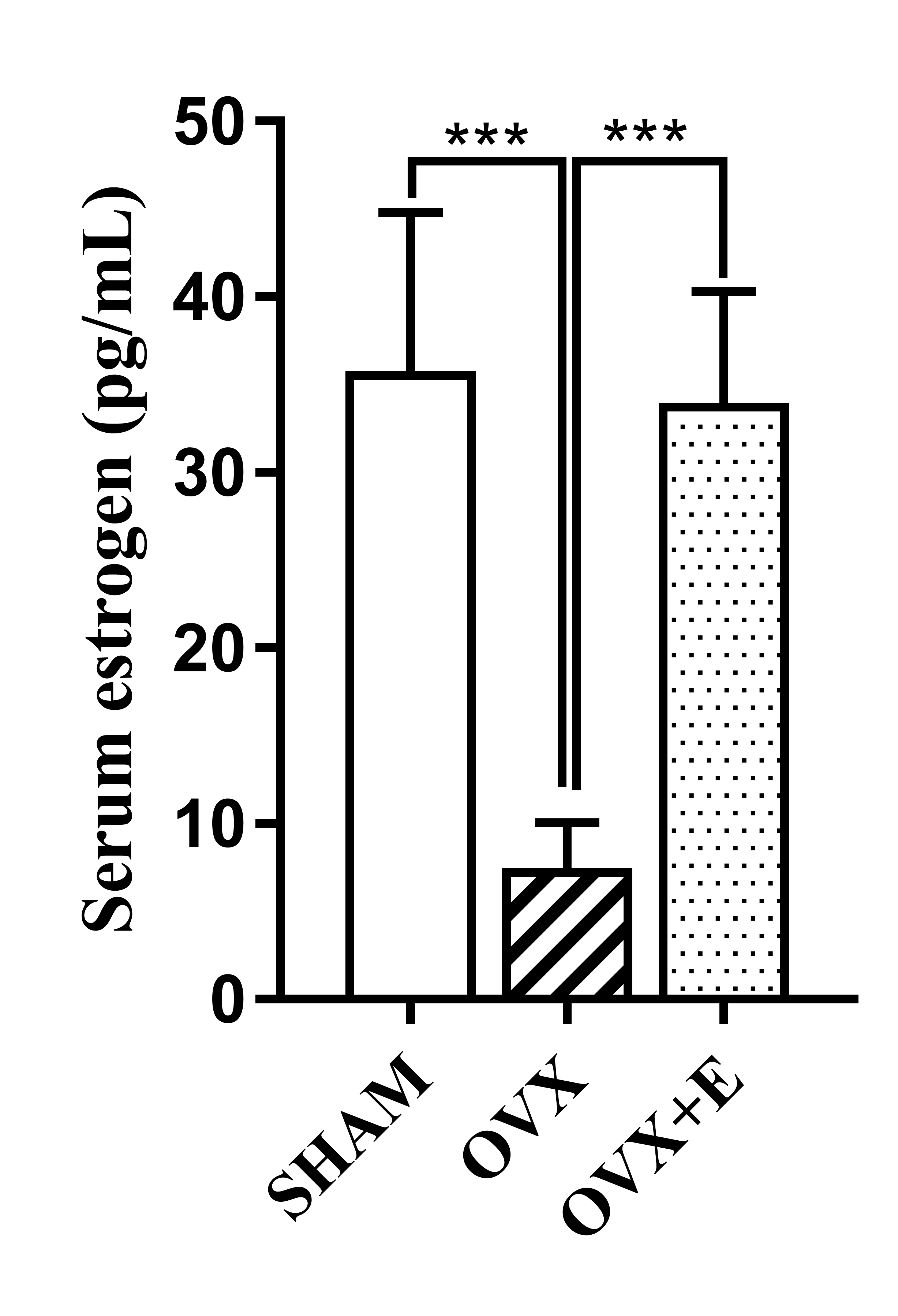

Supplement: Supplementary file 2 — Additional file 2: Figure S2. Comparative analysis of the concentration of serum estrogen between the SHAM group, the OVX group, and the OVX + E group. SHAM group: sham-operated group; OVX group: ovariectomized group; OVX + E group: OVX group treated with estradiol valerate. Data presented as the mean ± standard deviation, n = 10. * P < 0.05, ** P < 0.01, *** P < 0.001. [file 12944_2020_1279_MOESM2_ESM.tif]
