# Supplementary material for: The imbalance in the aortic ceramide/sphingosine-1-phosphate rheostat in ovariectomized rats and the preventive effect of estrogen
Source: Lipids Health Dis. 2020 May 19;19:95. doi: 10.1186/s12944-020-01279-7 (PMC7236922; doi:10.1186/s12944-020-01279-7)
Supplement: Supplementary file 3 — Additional file 3: Table S1. Primer sets of targeted genes. [file 12944_2020_1279_MOESM3_ESM.docx]

Supplementary Table 1 Primer sets of targeted genes

| Gene | Sequence (5’→3’) | |
| --- | --- | --- |
|  | Forward primer | Reverse primer |
| SPTLC1 | CTCCAAGAGCCTACATTCCTC | ACAGTCTTTGGTTGAGCAGG |
| SPTLC2 | TCCTGTCTCCACTGGGTGAG | GGATGTGTTCTTTGGTCAAGC |
| SPTLC3 | CCTCTTGATGTTCTCCTACAGC | TTTAGGCTCCCTCTGCATGG |
| LASS1 | TTCTAGCCAACTTCTGCCAG | ACAGCGTGGTTAAGTGCAG |
| LASS2 | GAAGCCAAGAAACTAGCCAAG | TCTCAACAGGATACAGCCAAG |
| LASS3 | TCTCCAGGCAGGGTCAGTTC | TCTGTGAGGTAGTAGAGCAG |
| LASS4 | ATGGTGTTGATGGTGCATGG | AGCTGTTACTCTGGTCAATGG |
| LASS5 | GAGTTAAGCTGTCTCCCAACC | GTGTCTATCCAGAGTGTTCCATC |
| LASS6 | GTCCCATCTGTGTCCCTTTAC | CTGTGTGAGGATCTTGCATG |
| nSMase | GGATATCTACTTCGCACACCG | GTCCACTCTTTCAGTAGGCAG |
| aSMase | CATAGCTTGTGGTCACGGCC | CTCAGGTAGATCAGCGATAC |
| Asah1 | CACCATGTCATTCACCTTTGC | CCATTTCCAGAGTCAGAGGTC |
| Cerk | CCCTGACATTCCCAAGTAG | CCACTGTCTAACCTTTACTCC |
| Sgms1 | GCGAAATTAACGGCATGATCC | TCGGAGAACAGTTGAAGTGC |
| Sgms2 | TTCCTTGCTGTTTCTCCTGG | GAAGCTGTGCCTTTGGTTAC |
| SPHK1 | AGCTGTCACCCATGAATCTG | GCTCCTGTATTTCTCACTCTCC |
| SPHK2 | CAGCTCAGCTTTCACCCATC | CCTCTATTGTCACCCAGTCTTG |
| S1P1 | AACTAGCAGGCTGTTGACTG | GAACACATGAGGAATAAGGAAC |
| S1P2 | TCTTCCCTTTCCTTCTGTGTTC | AGTTTTCTCACCAGGAAGCC |
| S1P3 | GGATCATTGGCCTATCTGTCTC | CTCAGCCCAGCACTTGCATG |
| GAPDH | TCTTCCACCTTTGATGCTGG | CTCTTGCTCTCAGTATCCTTGC |
